# Supplementary material for: Super-Suppression of Long-Wavelength Phonons in Constricted Nanoporous Geometries
Source: Nanomaterials (Basel). 2024 May 3;14(9):795. doi: 10.3390/nano14090795 (PMC11085507; doi:10.3390/nano14090795)
Supplement: Supplementary file 1 [file nanomaterials-14-00795-s001.zip › nanomaterials-2943586-supplementary.pdf]

Super-suppression of long wavelength phonons in constricted nanoporous geometries

Supporting information

Table S 1: Table of geometric features for porous systems with spherical pores. r is a measurement of the radius of the pore, d measures the distances between pores lengthwise (black double pointed arrow in blue geometry), and L measures the distance from the center of one pore to the center of the pore above (black line in blue geometry).

Pores	r (nm)	d (nm)	L (nm)
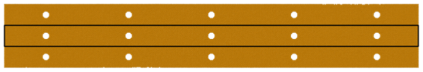	0.87	20.0	5.44
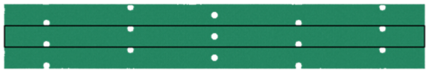	0.87	20.0	5.44
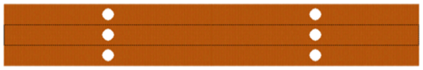	1.63	51.0	5.26
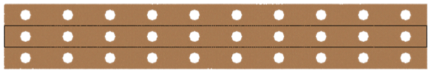	1.37	8.0	5.34
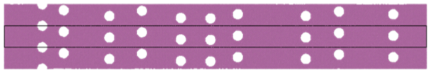	1.37	N/A	5.34
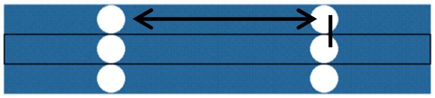	3.67	46.8	7.64

Table S 2: Table of geometric features for porous systems with spherical voids. The measurements of r, d and L are the same as shown in **Table S1**. For the measurements of d with asterisks, that indicates the distance has been measured from one cluster of pores to the next cluster of pores lengthwise.

Voids	r (nm)	d (nm)	L (nm)
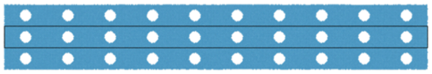	1.56	7.8	5.6
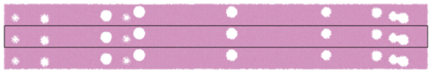	1.56	N/A	5.6
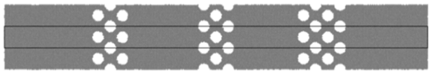	1.56	1.62*	5.6
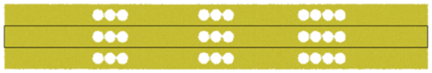	1.53	1.62*	5.4

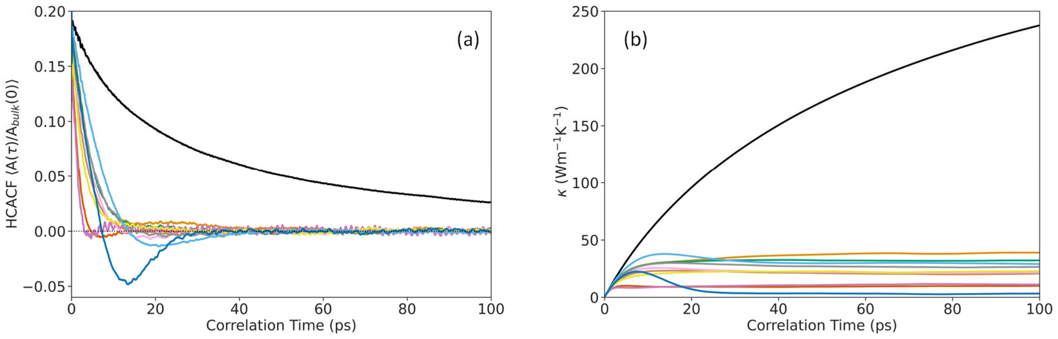

Fig. S1: A plot showing the full range of the pristine thermal conductivity and the thermal conductivity of the porous materials studied: a) the HCACF. B) The cumulative HCACF which sums up to the thermal conductivity of the different materials.
